# Effect of heavy metal exposure on human kidney cells

**DOI:** 10.6026/973206300211539

**Published:** 2025-06-30

**Authors:** Sahil Purushottambhai Patel, Charu Mishra, Versha Kumari, Sanket Patel, Kirti Nishant Vyas, Shruti Dalpatbhai Kalola

**Affiliations:** 1 Department of Health, GMERS Medical College & Hospital, Vadnagar, Gujarat, India; 2 Department of Physiology, Madhav Prasad Tripathi Medical College, Siddharth Nagar, Uttar Pradesh, India; 3 Department of Emergency Medicine, Max Hospital, Noida, Uttar Pradesh, India; 4 Department of Physiology, GMERS Medical College & Hospital, Vadnagar, Gujarat, India; 5 Department of Pathology, Dr N D Desai Faculty of Medical Science and Research, Dharmsinh Desai University, Nadiad, Gujarat, India

**Keywords:** Toxicity, kidney cells

## Abstract

Heavy metal cytotoxicity using lab-based cell culture of human kidney (HK-2) cells is of interest. The exposed cells contained lead (Pb), cadmium (Cd) and mercury (Hg) at dosages of 5 µM, 10 µM and 20 µM during incubation periods of 24 hours, 48 hours and 72 hours. Cadmium induced the maximum cell death through its dose-dependent pattern while exposing cells to 20 µM cadmium for 72 hours at 52.4%. Next in line was mercury which reduced cell viability to 60.8% and lead reduced it to 68.9% when cells were exposed to 20 µM for 72 hours. Under cadmium treatment at 20 µM the levels of ROS rose 2.3 times in the target cells. The cells experienced shrinkage of their size and developed membrane blisters through morphological transformations. This study reveals severe kidney damage because cadmium caused the worst impact on renal cell structure.

## Background:

The atomic weights and densities of natural heavy metals outweigh those of water five times or more. Heavy metals comprise zinc, copper and iron which have biological importance but also incorporate toxic substances like Cd, Pb and Hg that adversely affect human health during extended periods of contact [1, 2]. The kidneys face exceptional risk from heavy metal toxicity since their task includes both filtrating substances from blood and reabsorbing xenobiotics and excreting them [3]. Low-level exposure to heavy metals like lead, cadmium, and mercury can still affect kidney function [4]. Oxidative stress plays a key role in heavy metal-induced renal damage [5]. Combined exposure to multiple metals may increase health risks more than individual exposures [6]. The poisonous environmental contaminant lead disrupts calcium-dependent cellular operations in kidney cells and causes nephropathic changes involving tubular atrophy and interstitial fibrosis [7]. Research has shown that exposure to mercury with its two major forms of inorganic and methylated can harm glomeruli and tubules by both reactive oxygen species production and thiol binding mechanisms [8]. The scientific community requires additional controlled *in vitro* investigations to study how specific concentrations of heavy metals affect cell responses within their laboratory environments despite recent discoveries about heavy metal nephrotoxicity *in vivo*. The experimental investigation will evaluate the toxic properties of cadmium, lead and mercury on cultured human kidney epithelial cells. The research utilizes cell survival tests together with ROS measurement techniques and cell structure analysis to understand the toxic properties of these elements relative to their effects on renal cell health.

## Materials and Methods:

Under an *in vitro* experimental setup researchers evaluated the cytotoxic impacts of cadmium (Cd) and lead (Pb) as well as mercury (Hg) toward human kidney epithelial cells (HK-2 cell line). The HK-2 cells that researchers obtained from a certified cell repository required a tissue culture medium containing DMEM/F12 with 10% fetal bovine serum (FBS), 1% penicillin-streptomycin and incubation at 37° under 5% CO_2_. The cells received a plate seed density of 1 x 10^4^ per well and required overnight cell attachment time. Each experimental well received unique cadmium chloride (5 µM, 10 µM, 20 µM) or lead acetate (5 µM, 10 µM, 20 µM) or mercuric chloride (5 µM, 10 µM, 20 µM) treatment for durations of 24, 48 or 72 hours. The control plates contained only culture medium without the addition of any metal treatment. The MTT assay evaluated cell viability during the experiment. The addition of 20 µL MTT reagent solution (5 mg/mL) took place in each well before the cells received a four-hour incubation period. The formazan crystals were dissolved using 100 µL of dimethyl sulfoxide (DMSO) after removing the supernatant. The analysis measured absorbance at 570 nm in the microplate reader to determine viability based on control groups. The quantification of intracellular reactive oxygen species (ROS) levels at 2', 7'-dichlorofluorescin diacetate (DCFDA) fluorescent levels provided oxidative stress evaluation in this experimental setup. Cells were treated with 25 µM DCFDA solution by incubating them at 37° for 30 minutes after which they were observed using the 485/535 nm fluorescence excitation/emission wavelengths. Cell shape alterations in the experimental samples could be observed using an inverted phase-contrast microscope while taking pictures to document features like rounded cells, shrunk cells and membrane blebbing effectively. The analysis used one-way ANOVA while Tukey's post hoc test served for specific analyses. This analysis used three separate samples and the selected p-value remained below 0.05 for statistical significance.

## Results:

Exposure of HK-2 cells to cadmium, lead and mercury resulted in a concentration- and time-dependent reduction in cell viability. As shown in Table 1 (see PDF), cadmium exhibited the highest cytotoxicity, with cell viability decreasing to 52.4% at 20 µM after 72 hours. Lead and mercury also significantly reduced cell viability, reaching 68.9% and 60.8% respectively at the same concentration and time point. Compared to the untreated control, all three heavy metals caused statistically significant reductions in viability at concentrations ≥10 µM (p < 0.05). ROS generation increased proportionally with metal concentration, especially in cadmium-exposed cells. At 20 µM Cd, a 2.3-fold increase in ROS levels was observed compared to control (p < 0.01), while mercury and lead induced 1.8- and 1.5-fold increases respectively (Table 2 - see PDF). Morphological analysis confirmed cellular shrinkage, membrane blebbing and detachment at higher concentrations of all metals. These findings suggest that cadmium has the most potent cytotoxic and oxidative stress-inducing effects on human renal cells, followed by mercury and lead (Tables 1 & 2 - see PDF).

## Discussion:

Research examines how heavy metals affect human kidney epithelial cells (HK-2) through the representative heavy metals cadmium, lead and mercury where cadmium exhibits the strongest toxic effects. Scientific reports have already established cadmium as a toxic substance for the kidneys that builds up in renal tubular epithelial cells while causing oxidative stress and cell damage [1, 2]. The laboratory results that show decreased cell survival rates when using higher cadmium doses match previous findings from both laboratory and human subject experiments [3, 4]. Research indicates that the extended biological lifespan of cadmium and its interaction with metallothionein contributes to kidney damage through mitochondrial dysfunction combined with DNA fragmentation because it helps deliver the compound to renal tissue [5, 6]. The research data showed a 2. 3-fold elevation of ROS after cadmium exposure thus validating oxidative stress as a crucial factor in cadmium toxicity. The toxic effects of cadmium exposure on renal cell injury including elevated ROS levels and lipid peroxidation and impaired antioxidant defenses were documented in both Zhong *et al.* and Barrera-Chimal *et al.* research [7, 8]. The use of inorganic mercury in this study resulted in severe cytotoxic and oxidative stress effects. The chemical bond between mercury and the thiol groups in proteins and glutathione results in depleted antioxidant amounts which subsequently causes structural change within kidney tissues [9, 10]. The experiments indicated cadmium is slightly less toxic but the observed ROS increase along with morphological changes indicates major apoptotic and oxidative cellular mechanism activation. Zalups and Lash showed in past research that mercury causes kidney damage by damaging mitochondria and boosting cellular calcium entry [11, 12]. Lead demonstrated the lowest cytotoxic effects compared to other metal variants although historically known for its systemic toxicity according to study measurements. The higher doses of the substance led to meaningful cell death while initiating elevated ROS production levels. Lead toxicity affects the balance of renal calcium alongside metabolic dysfunctions before leading to tubular cell damage and fibrosis of the inter-stitium [13, 14]. Previous research documents that sub-chronic lead exposure can contribute to progressive nephropathy along with the moderate toxicity levels detected in this investigation [15]. Cadmium proves more toxic for HK-2 cells than mercury which in turn exhibits higher toxicity than lead according to the cellular uptake mechanisms of these metals and their affinity toward renal tissues. Most of the complexities which exist during systemic exposure and bioaccumulation as well as organ-level metabolism cannot be replicated properly through *in vitro* testing. The obtained findings demonstrate the necessity for enhanced government policies restricting exposure of heavy metals in the environment and workplace. Upcoming studies should aim to establish complete understanding of renal apoptosis mechanisms linked to metal exposure by investigating mitochondrial pathways as well as Nrf2-antioxidant response elements and autophagy mechanisms. Research into protective agents including chelating agents, antioxidant substances and modulating genes in combination treatment may lead to discoveries about heavy metal nephropathy prevention methods.

## Conclusion:

The *in vitro* tests establish that human kidney epithelial cells suffer dose- and time-dependent toxicity from cadmium and lead with mercury showing the highest toxic properties among these heavy metals. Oxidative stress markers combined with morphological cell damage show that reactive oxygen species (ROS) serve as primary pathways during heavy metal toxicosis of kidneys. The study shows how controlling exposure to heavy metals in environmental and occupational settings remains vital to defend kidneys from injury together with their subsequent health threats.
